# “I can never be in a room alone and not be on my phone.” Understanding smartphone addiction of young adults

**DOI:** 10.3389/fpsyg.2026.1744046

**Published:** 2026-08-03

**Authors:** Sandra Kröger

**Affiliations:** Social and Political Sciences, Philosophy and Anthropology, University of Exeter, Exeter, United Kingdom

**Keywords:** smartphones, addiction, availability, need to belong, platform design, young adults, focus group

## Abstract

The literature on smartphone addiction, particularly concerning young adults, is mostly quantitative and focuses on screen time and the self-assessment of whether digital users consider themselves addicted. The result is conceptual imposition and a lack of understanding of what produces smartphone addiction. This study takes a different, qualitative, and abductive approach. Eight focus groups were conducted involving 37 students aged 18–22 to explore how smartphones support and undermine wellbeing, with addiction emerging as one of the themes. The data were matched against the established six criteria of behavioural addiction. I find that five of the six criteria are widely met, thereby justifying the term ‘addiction’. The findings show that the multi-functionality of smartphones cannot account for smartphone addiction. Rather, it is a multi-layered process consisting of the psychological need to belong, platform design, and the social norms of constant availability and quick responsiveness that leads young adults into addictive behaviour, despite the feelings of guilt and stress that such behaviour creates in them.

## Introduction

While there are many advantages to smartphones, such as greater ease of communication, access to information and relaxing activities, organisation of tasks, and the convenience that comes from all these services being integrated in a single device, it is now established that they can also give rise to a number of disadvantages and health hazards. These include, but are not limited to, addiction, dangerous and/or prohibited use, aggressive behaviours, sleep deprivation, depression, anxiety, and low self-esteem (Billieux et al., 2015b). Addiction links many of these negative effects, and as such deserves particular attention. This article explores whether the smartphone usages students describe resemble addiction, and how we might better understand addictive smartphone behaviours.

The 2026 court decision against Meta certainly suggests that some platforms can make people addictive, and from a young age onwards. The available data point in a similar direction. In 2025, a World Health Organisation (WHO) study identified smartphone addiction as a public mental health concern in 54 countries.^1^ More specifically, surveys show that 82% of Gen Z acknowledge feeling addicted to social media or being dependent on it while 76% of Gen Z users find that they spend more time on their smartphones than they would like, which is not surprising given their average scree time is now 6 h and 27 min.^2^ Relatedly, 68% of students in a recent UK-based study thought that their phone usage harmed their academic performance.^3^ While in the USA, survey data from 2023 show that 56.9% of Americans admit that they are addicted to their phones, with 71% spending more time on their phone than with their romantic partner.^4^ Whether or not we should speak of smartphone addiction or use other terms is a contested matter to which I shall return in the next section.

This study focuses on young adults. They are more likely to lack the required self-regulatory capacity to control their phone use (Pokhrel et al., 2013) and are more vulnerable to the potential harms of heavy smartphone usage, not least because of their need to belong (Perrin and Anderson, 2019). It mostly draws on eight focus groups with university students aged 18–22, and a complementary quantitative survey.

Qualitative data, such as that presented here, is a much-needed addition to the existing quantitative work in the field. Because ‘addiction’ emerged as an unsolicited theme from the data rather than being deducted from theory, the risk of conceptual imposition is reduced and the validity of the findings increased. Qualitative data also play a crucial role in understanding smartphone usage and how smartphone addiction comes about. So far, what qualitative work there is on this topic has focused on the relationship of young adults with their phones and the role the device occupies in the users’ life (Fullwood et al., 2017; Harkin and Kuss, 2021), what motivates social media use (Throuvala et al., 2019), whether criteria of smartphone addiction are present or not without however seeking to understand them (Jameel et al., 2019), and what the barriers might be to move from ‘over-reliance’ to digital detox or dysconnectivity (Conroy et al., 2023). However, there is a lack of understanding of what it is that drives young adults into addictive smartphone behaviours (Fullwood et al., 2017, p. 354).

This study contributes to filling this gap. It generates novel insights by exploring the six established criteria of behavioural addiction and so improves our understanding of smartphone addiction. The findings show that whereas the multi-functionality contributes to dependency on the technology, it is the dynamic interaction between psychological needs, platform design, and social norms that create smartphone addiction amongst young adults.

The article proceeds as follows. In a first step, I review relevant literature around smartphone addiction and define the concept. As we will see, there is no consensus on whether there is widespread smartphone addiction or on what causes it, in so far as it does exist. The second section describes the research design and the methods used—eight focus groups with University students and a complimentary survey. The third section displays the empirical findings, zooming into the six symptoms of smartphone addiction, and analyses them. The fourth section reflects on whether we can indeed speak of smartphone addiction, answering the question positively, before moving on to discussing how we can understand the phenomenon. It understands smartphone addiction as a three-layered process, where individual psychological needs, platform design, and social norms interact and reinforce each other. The conclusion summarises the findings and wraps up.

## Literature review

There is no scholarly consensus as to whether we should speak of smartphone ‘addiction’ (Ryding and Kaye, 2018). Many scholars have adopted the term ‘addiction’ (Conroy et al., 2023; Kwon et al., 2013; Pontes et al., 2018; Samaha and Hawi, 2016) and have shown that some or all components of behavioural addiction are met, such as experiencing distress (Walsh et al., 2008) or conflict expressing in cognitive failures when one’s mobile phone is not available (Hadlington, 2015; Hong et al., 2020).

Whereas, initially, definitions of addiction focused on substance overuse, technical developments have led to a revision in the definition of addiction to also include behavioural addictions such as gambling or excessive smartphone use (Griffiths, 2005; Matar Boumosleh and Jaalouk, 2017). Drawing on Griffiths (2005), the established six symptoms of behavioural addiction are: salience, tolerance, mood modification, conflict, withdrawal, and relapse (Andreassen et al., 2017).^5^

In the present context, salience refers to being preoccupied constantly by one’s phone; mood modification relates to using it in order to reduce negative feelings; tolerance means gradually using one’s smartphone more in order to get the same pleasure from it; withdrawal expresses itself in suffering psychological and possibly physical distress if smartphone use is discontinued; conflict means sacrificing obligations and/or causing harm to important life areas because of the smartphone use, not least because of sleep deprivation; and relapse refers to trying to control the use of the smartphone without success. In the behavioural addiction literature, it is considered that if three or more criteria are met within a 12-month period, an individual can be defined as addicted.

Other scholars have questioned the validity of applying the terminology of ‘addiction’ to smartphone use, pointing to the over-pathologising of everyday life (Billieux et al., 2015a,b; Kardefelt-Winther et al., 2017; Ryding and Kaye, 2018). They argue, for instance, that ‘tolerance’ will play out very differently depending on the life situation of a user (such as their relationship status, whether starting or ending a job, moving house, age, etc.). Different factors will significantly influence usage without necessarily saying much about whether someone is addicted or not (Billieux et al., 2015b, p. 157).

Instead, these scholars propose to speak of problematic smartphone use (Horwood and Anglim, 2021), smartphone dependence (Billieux et al., 2008), nomophobia (Han et al., 2017; King et al., 2013; Yildirim and Correia, 2015) or other, similar, labels. The advantage of problematic smartphone use is that it avoids pathologising everyday behaviour, its disadvantage is that it is very broad, does not assume a specific mechanism, and so cannot explain said behaviour, and that it is conceptually vague, leading to inconsistencies in measurement. Smartphone dependence likewise avoids pathologisation but lacks clear criteria and so can relate to physical, psychological, or functional dependence. Nomophobia, by contrast, precisely captures anxiety that relates to not being able to access one’s smartphone or being without connectivity, and so is comparatively easy to operationalise. Its advantage is at the same time its disadvantage, in that it is too narrow to capture the different dimensions of smartphone addiction. It is fair to say that these terms are often used interchangeably by authors in the field (Cheng et al., 2025). The overall direction of travel seems to be towards acceptance of the term ‘addiction’ (Cheng et al., 2025), perhaps because it not only provides a strong theoretical framework, but also has public relevance and is easily understood.

Scholars have looked at different factors that might cause smartphone addiction, and more specifically social media addiction, mostly psychological factors such as maladaptive cognitions, limited self-regulation, loneliness, or depression, amongst others (Kuss and Griffiths, 2017). These factors in turn interact with Fear of Missing Out (FoMO). FoMO is the ‘pervasive apprehension that others might be having rewarding experiences from which one is absent’ (Przybylski et al., 2013, p. 1841; Hunt et al., 2018; Reer et al., 2019) and might be an important predictor of smartphone addiction. In turn, smartphone addiction may further increase the level of FoMO. A bidirectional influence between smartphone addiction and FoMO is indeed possible (Li et al., 2022). Other scholars have adapted a critical political economy approach and looked at deliberately addictive platform design to explain smartphone addiction (Zuboff, 2019). This research did not have a preconceived view of whether we can or cannot speak, of smartphone addiction in young adults, and what might cause it. Indeed, ‘addiction’ was not a concern initially and not directly addressed in the focus groups. However, in the qualitative data of this study, ‘addiction’ was one of the four themes that emerged from the analysis, suggesting that it is an important aspect of smartphone use. This finding provided an excellent opportunity to assess whether the existing definition of behavioural addiction can be applied meaningfully to smartphone usage by young adults. If we found significant evidence for smartphone addiction without having explicitly looked for it, then this would constitute a strong argument for smartphone addiction being present.

## Methodology

As has been pointed out, the existing quantitative studies not only neglect important aspects of smartphone addiction but are also to some extent self-confirmatory (Billieux et al., 2015b, p. 159). If one asks participants about addiction, one is bound to find some addiction-like symptoms. Similarly, if one asks individuals who self-identify as addicted to smartphones (Aksoy, 2018), the sample is not representative. This study took a different approach and followed an abductive research design, where bottom-up data generation and theory interact in a dynamic way (Friedrichs and Kratochwil, 2009, see below for what this meant in practice).

### Methods

This study draws primarily on qualitative data coming from focus groups that were used to explore and understand smartphone usage by young adults (see Appendix 1). Focus groups seek to elicit peoples’ experiences, understandings and opinions of things (Carey, 1994). They are a method ‘that capitalises on communication between research participants’ (Kitzinger, 1995, p. 299), with the interaction among participants adding a valuable dimension to the data collection that is absent from other methods, including one-to-one interviews (Kitzinger, 1995; Morgan, 1996), not least because they allow the researcher to observe the degree of agreement and disagreement among participants.

Some scholars worry that particularly sensitive topics can suffer from social desirability biases (Montoya-Weiss et al., 1998). I consider that there are no universally accepted norms in terms of desirable smartphone behaviour. There are also concerns regarding the limited validity of focus groups results (Kitzinger, 1994). I consider that this concern is attenuated by the high number of focus groups (8). The emergence of substantively similar agreements and disagreements in multiple focus groups leads to data saturation (Hancock et al., 2016; Francis et al., 2010) and thereby supports content validity (Kidd and Parshall, 2000).

Each focus group included open-ended questions based on a semi-structured guide. All the questions were kept intentionally broad and did not follow a specific theory or approach (see Appendix 1). Students were asked in very general terms what smartphone usage and availability looked like, and how participants thought it supported and undermined their wellbeing. The same questions were asked about social media, with some additional added and which specifically addressed the topics of FoMO, social comparison, and image editing, given those are prominent topics in the literature on social media addiction.

In addition to the focus groups, I also deployed a quantitative survey to generate some descriptive and complementary information (see Appendix 2). While the focus groups allow for a better understanding of how smartphone usage unfolds, the survey provides information about aspects that could not be covered in the focus groups due to time limits or because of participants potentially being less ready to share in the focus group. In that sense, they complement the data gathered in the focus group without seeking to validate it. For the purpose of this article, only that data is used that speaks to one of the six addiction criteria. More concretely, this refers to daily screen time (tolerance) and how easy it was for students not to use their smartphone for a day (withdrawal). The answers to a third question—which apps participants most use—are part of the discussion that follows the display of findings.

### Process

Prior to running the survey and holding the focus groups, ethics approval was obtained from the University of Exeter. Participants were recruited at the University of Exeter by means of on-site posters and mails sent to all the students from the then existing Colleges, with the selection criterion being that participants had to be undergraduate students. Interested students received an information sheet which summarised the project and its aims, explained what participation would entail as well as what would happen to the data. Once students confirmed their interest, they received a consent form in which they confirmed they had read and understood the information sheet and consented to taking part. Once they returned the signed consent form, they were allocated to a focus group and sent the link to the survey which they were asked to complete prior to the date of their allocated focus group. After completing the focus group, students were paid £10 for their participation. Eight focus groups were conducted in March 2022, with each group consisting of four to five students.^6^ The focus groups lasted approximately 90 min and were held online and audio-recorded via zoom, with the transcription function activated.

### Analysis

The zoom transcriptions were reviewed twice by the author to ensure the quality of transcription^7^ before being input into NVivo. To ensure participants’ anonymity, all identifying information was removed from the transcripts, and participants were allocated identification codes. These codes included the unique participant number (P#) and the focus group (FG#) in which they participated (e.g., P1, FG1). They are used in the findings section to evidence the source of quotations. Thematic analysis was applied by three coders, including familiarisation with the data, generation of initial codes, reviewing of codes, searching and reviewing themes, and defining and naming themes (Braun and Clarke, 2006). The author of this study initially coded the transcript of one focus group line-by-line, with codes generated inductively from the data rather than from a pre-existing framework. Segments of text ranging from a single word to several sentences were assigned codes that captured their meaning. To enhance reliability, two additional coders independently reviewed the transcript and the initial coding framework. Codes were discussed collaboratively with some codes merged and others added, leading to an agreed coding scheme. On this basis, the remaining transcripts were each coded by the additional coders, after which they shared the coded transcripts and emerging interpretations with the author. New codes were discussed collaboratively, and we decided which new codes to keep and which to merge, after which texts were read again to consistently add the new codes throughout. On this basis, related codes were grouped into broader categories based on conceptual similarity which were then developed into themes. Four themes were named and defined, after which the author immersed herself in the theoretical literature around the addiction theme. With the six established criteria of addiction at hand, the codes relating to the theme of addiction were linked to said criteria. As we will see, some of the criteria found more support than others, which is not surprising. This back-and-forth process between theory and data is what defines the abductive, pragmatic research strategy.

## Findings

### Descriptive information

Participants consisted of students (*N* = 37, 27 female, 10 male) aged 18–22 (*M* = 20.05) coming from across the University.^8^^,^^9^

## 6 indicators of smartphone addiction

### Salience

Virtually all the participants said that they were *constantly* preoccupied with their phones, not least because they were an indispensable tool which facilitated *every* aspect of their lives. Amongst other purposes, they used their phones for communication, as a diary, banking, shopping, buying tickets, navigation, entertainment, and wellbeing. One student explained: ‘It’s my alarm in the morning, what I do when I’m bored, how I communicate with people, how I find out where my friends are. So, it’s almost for every aspect of my life which is probably why my screen time is so high’ (FG6, P27), illustrating how diverse routines converge into a single device.

Participants noted the temporal consequences of their usage and thought that they spent more time than they were comfortable with on their phones. Students commonly said that it was the smartphone’s multi-functionality that led them to easily being sucked into the world within their phones, often making them feel like they lost hours of their day. As one student put it: ‘My phone sends me my screen report and it’s 4 1/2 h or something. You’re like I do not actually know why I spent that much time on any of these things. It just disappears’ (FG4, P18), reflecting the device’s capacity to capture and sustain attention.

This functional centrality appears to erode users’ capacity to operate independently of the device. Most participants thought that they had either forgotten or never learned how to go about these activities without their phones. A widely shared sentiment was that one would be lost without it, reflecting a deep dependency. As a result, the absence of their phones was equated to feeling handicapped: ‘Two months ago, my phone just died out of nowhere… and I realised how much of a handicap it was to not have it. I had an important class I couldn’t miss… I didn’t know which bus I had to take. It was quite far away and so I had to go back to just writing out instructions which times the bus is coming and find an old watch’ (FG2, P8). In this sense, salience reconfigures competence, where absence of the device disrupts basic functioning.

Importantly, salience is reinforced through social norms embedded within smartphone communication. Most students pointed to social pressure to make themselves available and respond to their friends’ messages and posts quickly: ‘I respond quite fast to things, and I kind of expect everyone else to, and when they don’t, I find that hard. I’m like, oh, is something wrong, he hasn’t replied to me’ (FG5, P23). This social expectation to respond quickly was called a ‘daunting task’ by one and a ‘toxic habit’ by another participant and a third explained how it ultimately is detrimental to her wellbeing: ‘The connectivity thing like you’re available 24/7, which is nice ‘cause you can reach out to people. But then as soon as I don’t answer someone’s message for an hour or something, I feel genuinely guilty about it. And if I know, for instance, that friend isn’t doing well at like another uni or like in another country, I feel like I constantly need to reach out to them and then my smartphone becomes like a point of stress rather than a point of like wellbeing’ (P30, FG7). Another student admitted: ‘I hate having to respond to messages. And I get this massive shame that there’s so many messages I haven’t answered. And it gets to the point where I feel bad if I do respond because it’s been too long’ (FG6, P26). Similar pressures were felt with regard to social media posts, where students felt as if they had a responsibility to respond to, comment on, and like the social media posts of their friends to maintain their friendships: ‘I have to keep up with all of it and look at the posts. And even if you try to look at everything, there’s always gonna be more’ (FG8, P36).

The perceived pressure to remain available and to respond quickly to messages and social media content operates as a form of social regulation. The anticipation of others’ responses further intensifies this dynamic, creating reciprocal pressures that sustain constant monitoring of the device. Consequently, salience emerges not only from individual habits, but also from collectively maintained norms of connectivity and responsiveness.

Overall, salience is constituted through users’ constant preoccupation with their phones, and the device’s integration into everyday practices which reconfigures competence. It is maintained through social norms of connectivity and responsiveness.

### Tolerance

Admittedly, this study did not measure longitudinal increases in smartphone usage to get the same pleasure from it. Nevertheless, the data provide evidence of tolerance. Self-reported screentime in the survey was between 5 and 7 h per day for 48.9% of the participants, with 11.1% exceeded 7 h per day.

Indeed, one of the biggest concerns that most students had was the amount of time that they spent on their phones. Students were consistently shocked and frustrated by high and ever higher screen time or the disappearance of hours in the day. As one student put it: ‘I don’t use it for anything that’s productive. I was five hours on social media, and I could probably be doing something a bit better with my day’ (FG2, P8). Another student said: ‘It kind of scares me how I spent 3 h on my phone already today. What was I doing all that time? I mean it is scary how much time goes’ (FG2, P7). The perception of ever higher screen time, coupled with difficulty accounting for how time was spent or it being characterised as unproductive, points to a gradual desensitisation, to extended baseline consumption and diminishing returns. This is consistent with tolerance processes, where increased exposure is required despite reduced subjective reward.

Feelings of guilt and frustration about the amount of time wasted further underscore this dynamic. Participants felt guilty about not dedicating their time to something they themselves thought of as more productive or valuable, such as studying or reading: ‘I guess I do judge myself when I use my phone a lot because as I said, I do not use it for anything that’s productive’ (P8, FG2). Another student offered that ‘it just blows into my time where I am genuinely relaxing, so I feel like looking at my screen time and that makes me feel really guilty to figure how much time I have been wasting’ (P4, FG1). The moral framing indicates an awareness that increased usage does not correspond to increased satisfaction, while the persistence of high usage despite the negative evaluation suggests that engagement is no longer governed rationally. Overall, tolerance in smartphone usage is about high screen time and the expansion of usage duration, coupled with a shortened satisfaction cycle and feelings of guilt.

### Mood modification

Mood modification played an important role in students’ smartphone usage, indicating that the device plays a central role in emotional self-regulation. Many participants mentioned seeking to escape loneliness and using their smartphones in an attempt to relieve stress. Pretty much all the participants turned to their phones when bored and for procrastination, leading them to purposelessly browse and scroll.

There was a broad recognition of the phone being used when feeling lonely and to escape loneliness. As regards the former, one female student offered ‘I think it’s ‘cause like you are on your phone, so you are already alone’ (P21, FG5). Another student, when speaking about social comparison on social media, suggested that ‘with friends, sadly, I do think it’s primarily negative, but I’m just basically comparing the fact that they are happy and I’m lonely probably when I’m looking at it’ (P23, FG5). Here, smartphone usage is reflecting feelings of social isolation. As regards the latter, social media is also mobilised as a strategy to escape loneliness. As one participant said: ‘So I live alone, so I do not have any roommates this year, and I guess I like being able to have the option to go on my phone so I’m not just eating a meal in silence, just with myself and I can FaceTime people or even just, yeah, it makes you feel less lonely’ (P8, FG2). Others commented on how they had used social media to find a community of like-minded (or like-sized) people: ‘I found like a positive community. I do not really use that so much anymore. But when I did use it, there was a lot of, you know, just like tips and advice that for me were like really healthy, and also just that sense of connection and not being alone in a specific situation’ (P35, FG8). Here, smartphone usage provides a sense of social connection.

Furthermore, many participants said that they used their phones to relieve stress, either to avoid work (procrastination) or to relax after a period of study. Many students referred to the mindless scroll, specifically the infinity scroll on social media platforms, that they often find themselves preoccupied with: ‘I do not want to do the work I actually have to do because [the mindless scroll] is the easier option’ (FG4, P16). In such cases, students consumed high amounts of content on the infinity scroll while essentially turning off their brains. As regards post-study relief, statements such as ‘I feel stressed after studying, then I will probably go to Instagram or YouTube to find some light baking video so that I can learn how to bake a cake or find new recipes’ (FG3, P12) or that social media serves ‘as a means to relieve my stress after studying for a long time’ (FG3, P12) were common. A female student said that she ‘tends to meditate before I go to sleep or in the morning. That helps me keep my sanity with the hecticness of uni. Also music, I think, really boosts my mood. I have the habit of walking to places while blasting music on my headphones, and that boosts my confidence’ (FG5, P22). Here, the device allows passive consumptions as a form of short-term relief from stress.

Students commonly said that they used their phones most when bored. In such situations, they found themselves preoccupied with the ‘mindless scroll’: ‘It’s like if you have not got anything specific [to do], we are just going on our phone because it’s a nice way of passing the time. It does not feel like a lot of pressure to do anything on your phone. You go on there to waste that time’ (FG6, P25). Or, as another student put it, ‘when I’m like bored and alone I just sculpture in social media’ (P1, FG1). Here, the device is used as an easy and pressure-free way to fill time and which provides distraction.

Finally, students used their phones to make themselves feel better. In other words, the phone was framed as a reward mechanism. Students liked ‘watching those funny videos on Instagram. So yeah, when you laugh, you feel better. Plus, you can send those, like the best ones, to your friends and what they like. And it’s just, again, a bit of an interaction by laughing together basically’ (FG3, P13). Here, mood medication relates to creating positive affective states.

Many participants realised, however, that resorting to their phone did not have the intended effect. As one student put it: ‘I felt like it’s a break, and it’s just not a break, and it’s just not relaxing your brain at all. [It] tires you out’ (P16, FG4), indicating that despite being aware of the limitations of smartphone-mediated mood regulation, students used it for precisely that.

Overall, mood modification means that the phone was used to regulate emotional states, by seeking to escape negative feelings or by creating positive ones, as well as for procrastination.

### Conflict

Smartphone usage created conflict in two important areas: university work and sleep. In the academic domain, participants reported that they often used social media in the in-between times of the day, such as between lectures or seminars, and acknowledged how detrimental this can be: ‘Practically, it is also a massive time waster and a massive distraction quite often, especially if you’re trying to do uni work or proper work or you know, stuff like that’ (FG2, P7). A few students mentioned that they had missed lectures or appointments due to their social media usage because they ‘can find it very easy to just lose track of time’ (FG8, P37). Students knew that that they were supposed to be focusing on their studies during daytime, whether it be reading or attending lectures/seminars, and yet smartphone usage led to tangible disruption of responsibilities, not least through the mindless scroll. Users’ intentions and their actual behaviours were thus misaligned, with students experiencing a conflict between the demands of academic study and the immediate, low-effort gratification offered by smartphone usage.

Sleep represents the second major site of conflict. Almost all the participants said that their sleep had been affected by prolonged social media usage prior to going to bed, while in bed, and during the night when waking up.

So far as social media consumption before bedtime was concerned, students rationalised device usage when time felt insufficient for more meaningful activities: ‘By the time I walked back from work and ate something, it’s only like an hour and a half or two hours prior to going to bed. So, it doesn’t feel like I can do anything meaningful with the time. So, I just end up going on my phone’ (FG5, P24). However, usage often extended beyond the initially intended time, delaying bedtime: ‘If I was to look at my phone, I can honestly be up for like two hours watching videos and that’s it. That’s when it affects me because you think, oh, it’s easier to go on my phone now and tire myself out by watching something boring like someone repainting a room on YouTube’ (FG6, P26). A couple of participants mentioned that they believed reading before bed would be better for their sleep. However, they struggled to break the habit of automatically checking their phones.

As regards smartphone consumption once in bed, some students reported that it was a routine to look at their social media accounts while lying in bed, often meaning to be only 20 min, but regularly turning into hours. One student said that she and her flatmates had a running joke that they would ‘all agree to go to bed and then two hours later we are still sending each other tik toks’ (FG6, P27). Thus, social dynamics contribute to extended engagement and normalise delayed sleep.

Finally, some students struggled with social media consumption when waking up in the night. For instance, two students reported that they regularly wake up around 4 a.m. and struggle to fall back asleep: ‘I will wake up at 4 in the morning and then at that point if I check my phone, I somehow end up on social media’ (FG7, P30). Both students considered their smartphone usage before sleep to be the cause of their sleep disruption, often accentuated through more smartphone usage during the night.

Overall, conflict materialised in the disruption of students’ academic work and sleep as they often prioritised social media over intended tasks and lost track of time. Crucially, this conflict persisted despite users’ insight into its negative effects.

### Withdrawal

None of the participants could imagine being without their smartphones for extended periods—anytime between a couple of hours and a couple of days. These findings are corroborated by the survey data: only 11.1% replied that they agreed or strongly agreed that it would be easy for them not to use their smartphone for 24 h whereas 80% disagreed or strongly disagreed.

The idea (or rare reality) of being without their smartphone created significant anxiety amongst all students. This could be generalised anxiety or context-specific anxiety. Generalised anxiety emerges in relation to the mere possibility of being disconnected: ‘I feel a bit anxious if I see the battery percentage going down. If it’s at 2% or 5%… I don’t know what to do without it … I get worried if it’s not there’ (FG3, P11). The student explained that this anxiety is rooted in feelings of being disconnected from the world and peers, a feeling shared by many students. Without the device, or even just the threat of potentially being without it, they reported feeling anxious about not being able to do their normal daily activities. This anxiety illustrates that withdrawal manifests in is a preoccupation with potential inaccessibility as well as actual absence of the phone.

As regards context-specific anxieties, ‘emergencies’ and ‘security’ often came up. Many students mentioned that they were worried that they might not be available or would be out of reach should someone—a friend or family member—need their help: ‘I want to be there for people if they are going through a tough time to listen and to give advice when I can’ (FG4, P19). This worry reflects how withdrawal is entangled with perceived social obligations, where the absence of the device compromises the user’s ability to live up to those perceived obligations. Furthermore, particularly female students also related the presence of the phone to their own (feeling of) security when walking home at night: ‘I think also for safety when I go out and walk back in the dark. I do not feel safe as a woman walking on my own. I do not feel safe. So having my phone with me, I tend to call someone, or I like to have it with me to have that option. I feel a bit safer if at least you can contact other people if something were to happen (FG8, P34). Unfortunately, this anxiety is rational. These different anxieties result in a near-constant presence of smartphones with users.

Overall, withdrawal means the inability to imagine being without a smartphone. The absence or potential loss of access triggered both general anxiety about disconnection and context-specific anxiety related to social obligations and safety, reinforcing the constant presence of smartphones.

### Relapse

Against the best intentions of students to try and limit their smartphone usage, there were many reports of these efforts regularly failing, showing that relapse is a central mechanism in smartphone addiction. The different techniques of withdrawal that students used, be it the temporary uninstallation of apps or the physical removal of the phone, were always short-lived.

Several students mentioned that during exam time, they will uninstall select social media applications: ‘My priority would be studying … I usually like to uninstall other social media accounts when the exams or coursework deadline is near’ (P12, FG3). While this strategy temporarily reduced engagement, students invariably reinstated usage after a certain period: ‘I often just uninstall things because I feel I don’t know about how long I’ve spent on them. But then you do get to a point where you’re like, oh, this and this is going on, and I feel like I’ve missed out on that. But that’s only when something has happened that you have missed out on, and that makes you re-download it’ (P28, FG6). The same result (of relapse) was reported by a student who used an out-of-sight-technique: ‘If I need to concentrate really heavily on say uni work, I will go downstairs and I’ll hide my phone in a kitchen drawer and just remove it from my life and my space. But a certain amount of time will pass, and I will come to really miss it and be running down the stairs to check it, so it never really lasts long’ (P20, FG5). Two women said that even with flight mode on, they still feel compelled to check their phones for the notifications they know are there as soon as they turn off flight mode. These strategies, therefore, provided only transient interruption, with students experiencing strong urges to re-engage.

The most frequently mentioned reason for relapse was the social expectation of being available: ‘I put it into flight mode, and I get called out for so many times by my friends and family “why am I not so available”?’ (P10, FG2) Indeed, one expectation that comes with having a smartphone is that of being constantly available while unavailability is socially frowned upon. This indicates that relapse is not purely intrapersonal, but is socially mediated, sustained by social norms around permanent connectivity and quick responsiveness. While students described social expectations and the pressure they create as ‘toxic’ and made wishful thinking statements about leaving the smartphone behind, or wanting an escape, they were unable to enact such changes in a lasting way.

**Figure 1:**
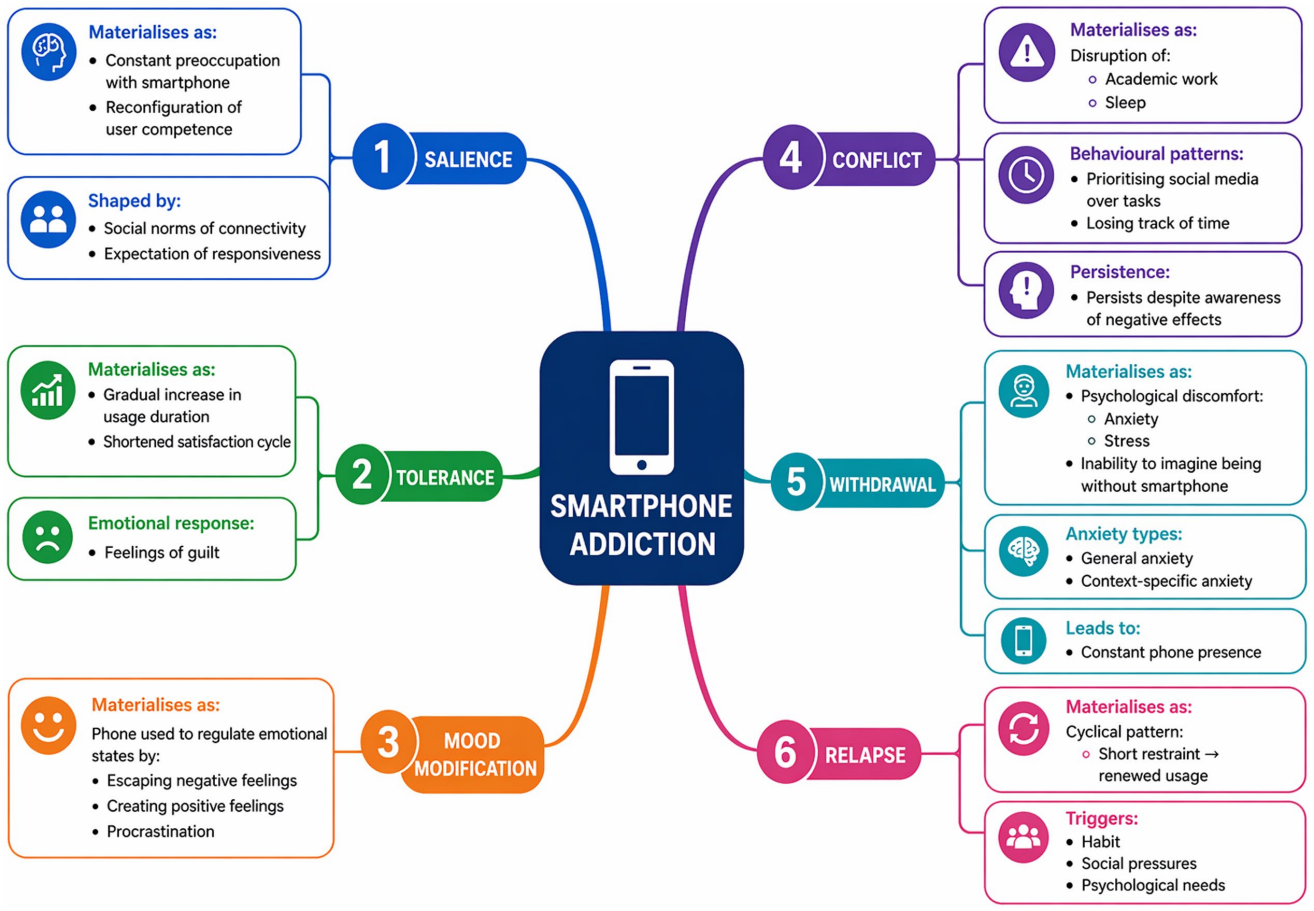
Criteria and mechanisms of smartphone addiction as emerging from the material, mindmap produced by ChatGPT.

Overall, relapse means that brief periods of restraint are followed by the reinstatement of device/app engagement, triggered by a combination of habit, social and psychological pressures (Figure 1).

## Understanding smartphone addiction

This section returns to the two main questions of this study, namely, can we speak of smartphone addiction amongst young adults, and how can we understand smartphone addiction?

There are clear traits of smartphone addiction as per the six criteria of behavioural addiction amongst almost all the participants of this study (see Harkin and Kuss, 2021 for similar results).^10^ Indeed, this is the vocabulary that many of the participants used for their smartphone usage. The multi-functionality of smartphones and strongly felt social pressure to make oneself available led to a high salience of smartphones in their lives. Tolerance materalised in high usage patterns. Mood modification played an important part, with escaping loneliness, boredom and stress relief figuring highly, and leading to purposeless scrolling. Conflict acted as a disruptive mechanism for many with regard to academic responsibilities and sleep. As for withdrawal, when without their phones, students felt handicapped and experienced anxiety. Relapse showed that attempts to be without their phone were only ever short-lived, despite students showing awareness that their smartphone usage had detrimental effects on them (Conroy et al., 2023; Fullwood et al., 2017; Harkin and Kuss, 2021). These different mechanisms do not operate in isolation but interrelate dynamically and create a self-reinforcing loop.

Some might consider that the high screen times is down to the multi-functionality of phones, making it more difficult to distinguish ‘addiction’ from ‘necessity’ or ‘dependence’ (Harkin and Kuss, 2021; Kuss, 2017). In a world where the smartphone allows you not only to communicate (interactional features), but also to pay, bank, wake you up, navigate to places, organise your day, and take pictures (functional features); where it lets you watch films or sports, listen to music and podcasts and play games (recreational features), and gives you access to information and news (informational features), it is increasingly difficult not to have an elevated screen time (Fullwood et al., 2017).

However, while the multi-functionality of the smartphone can lead to dependence, it is not what leads to the high screen times that we can observe in young adults. Tellingly, it is apps such as snapchat, Instagram, what’s app, tik tok and the like that were ranked highest in terms of usage in the survey, whereas apps such as google, chrome, safari, netflix, spotify, etc.—non-interactive apps—were generally ranked lower, implying that it is the interactive apps which lead to the very high screen time and thus the addictive behaviour (Reiner et al., 2017). This could suggest that the multi-functionality of the smartphone creates dependence whereas the interactive apps create addiction. Indeed, some have suggested that young adults are not necessarily addicted to the technology itself, but to the idea of being connected with people (Kuss and Griffiths, 2017) and so argue that ‘smartphone addiction’ misidentifies the unit of analysis. Others argue that addiction frameworks risk over-pathologising everyday behaviours (Billieux et al., 2015a; Kardefelt-Winther et al., 2017), particularly when the multi-functionality of smartphones necessitates regular engagement with the device.

While it is true that the findings of this study confirm the functional dependence on smartphones, the data also support an account of addiction. The cyclical and self-reinforcing nature of participants’ behaviours in particular suggests that more than mere habit or functional dependence is at play. The way students were constantly preoccupied with their phones, even when not looking at them, and how this led to gradual increases in usage duration to achieve the same affective result while experiencing feelings of stress and guilt for usage patterns; the ways students used their phones mostly for emotional regulation, and how the high usage dosages led to disruption of academic responsibilities and sleep, and the repeated nature of that despite an awareness of the negative effects; and, finally, the high levels of discomfort and anxiety felt at only the idea of withdrawal (Abeele et al., 2018; Vorderer and Klimmt, 2020) as well as regular experiences of relapse all testify to the addictive patterns in usage behaviour. What is more, participants experienced smartphones ‘as an extension of my arm’ (P20, FG5, see Throuvala et al., 2019, p. 171; Konok et al., 2016; Konok et al., 2017 for similar findings), thereby taking on physical traits. Being without a smartphone, or even switching it off, becomes an unthinkable eventuality. Rather than dismissing the concept of addiction, there are therefore good reasons to develop the concept in ways that take into account the context in which addiction operates.

This finding leads us to the second question: namely, how can we understand smartphone addiction? So far, the literature has concentrated on psychological factors, not least higher neuroticism and lower conscientiousness (Carvalho et al., 2018; Marengo et al., 2020). The findings of this study suggest that we need to enlarge our understanding of smartphone addiction and understand it as a phenomenon that emerges from the interaction of psychological factors, not least the need to belong, with platform design, and social norms.

The *need to belong* (Baumeister and Leary, 1995) means that humans have a fundamental motivation to form and maintain strong and stable relationships so that they feel accepted and valued, making it a basic psychological need. The need to belong is particularly felt by young adults (Mai et al., 2015) because of ongoing processes of identity formation, heightened peer importance, and sensitivity to social evaluation.

Social media gratify this need (Ha et al., 2015; Mai et al., 2015). They allow young adults to interact with friends, family, and others 24/7. They offer the possibility to constantly keep constantly informed about others, and constantly inform others about themselves, as well as to provide and receive feedback. Indeed, amongst the participants of this study, the desire for connection seemed to underpin much of their engagement with their phones. Students’ anxiety about the possibility of being disconnected reflects a concern with maintaining social inclusion and visibility, and unsurprisingly, FoMO played an important role in smartphone usage, regularly leading students to check what their friends are, or had been, up to.

As well as gratifying the need to belong, the *design of digital platforms* further amplifies it, to the point of engendering addiction (Alter, 2017). As some argue, addiction is designed into the system for commercial profit, with high user engagement translating into a high number of data points which can be mined and sold to third parties for profit (Zuboff, 2019).

Social media apps, in particular, are designed to capture users’ attention, command continual engagement, and drive users to seek constant gratification by means of intermittent variable rewards (linking a user’s action to receiving a variable reward). Updates, notifications and reminders suggest to users they should engage (Harkin and Kuss, 2021; Kuss et al., 2018), while badges, likes, views, streaks, levels and points, matches, and colour schemes, are stimuli which give dopamine shots to app users, making them crave for more (Ytre-Arne et al., 2020; Alter, 2017; Walz and Deterding, 2015), while infinity scrolls keep users engaged beyond their initial intent.

Amongst the participants, there was an awareness of this addictive design—roughly half of the participants of this study commented on social media being specifically designed to keep users hooked. Students were particularly aware of how algorithms sought to keep them engaged by showing content geared towards individual users when speaking of ‘mindless scrolling’ and the ‘infinity scroll’ that they linked to apps such as tik tok. But the awareness was not strong enough to overcome addictive behaviours.

The addictive design of social media apps and permanent connectivity, combined with the need to belong, work together to create *new social norms*—those of constant availability and quick responsiveness. The norms are internalised even in the absence of explicit coercion (see Foucault, 1977), and without anyone having declared them a rule or policy. They are a product of the mere possibility to be connected everywhere, all the time (Vorderer and Klimmt, 2020). Non-compliance with the norms, by contrast, produces guilt and social anxiety. As a result, young adults feel obliged to make themselves available and respond quickly to messages (Conroy et al., 2023; David and Roberts, 2017; Turkle, 2017), so as to avoid feared social repercussions and feelings of guilt for not having been available.

As we saw in the data, peer expectations led students to make themselves more available than they would normally be happy with, and also led to relapse (see also Olivencia-Carrión et al., 2018; Throuvala et al., 2019). While participants were aware of the stress created by constant availability, they still prioritised availability despite later feeling guilty for having wasted too much time on their phones. That they chose to make themselves available despite the stress it clearly produced for them can be linked to their need to belong. When choosing to opt out of constant availability, students felt they had let their friends down which in turn led to feelings of guilt and related stress.

## Conclusion

This study addressed whether young adults’ smartphone use can be meaningfully understood as addiction and, if so, what drives addictive behaviours. The findings provide considerable support for the presence of addiction-like patterns, with participants exhibiting five of the six core criteria of behavioural addiction. However, the findings suggest we need a more nuanced interpretation of ‘addiction’ than that of an individual pathology: namely, that smartphone addiction is best understood as emerging from the interaction of psychological needs, platform design, and social norms. Such an enlarged conceptualisation of smartphone addiction does not do away with individual differences, such as personality traits or coping styles. It is more about moving on the conversation from focusing exclusively on individual factors towards also integrating systemic and societal factors. A logical implication of such integration is the need for structural interventions, not least the redesign of digital platforms and a critical re-evaluation of social expectations of constant availability. Without such changes, efforts to reduce smartphone addiction are likely to remain limited, as individuals continue to operate within systems that actively incentivise and normalise compulsive engagement.

Despite this study’s strengths—notably, the way addiction emerges as a theme rather than being imposed through theory, and the insights into the multi-layered nature of smartphone addiction—some limitations must be acknowledged. The sample is relatively small and based on self-selection, which may bias the findings towards individuals who are more reflective of their smartphone use. Additionally, the qualitative data of this study do not allow for generalisation. Future research should combine qualitative and quantitative approaches to further validate these findings. In particular, the multi-layered model where psychological needs, platform design and social norms combine to form addictive behaviours should be given attention. While the psychological need to belong is well established, we need to more about how addiction is engineered through variable rewards system, and how technology works to create new social norms which in turn reinforce psychological needs.

## Data Availability

The raw data supporting the conclusions of this article will be made available by the authors, without undue reservation.

## Footnotes

1 For the purpose of open access, the author has applied a Creative Commons Attribution (CC BY) licence to any Author Accepted Manuscript version arising from this submission.

2
https://sqmagazine.co.uk/smartphone-addiction-statistics/

3
https://www.thetimes.com/uk/technology-uk/article/average-young-person-25-years-phone-screen-time-hwt76mnpq

4
https://explodingtopics.com/blog/smartphone-addiction-stats

5 Griffiths later argued for ‘loss of control’ as a seventh component, and this component is included in the problematic mobile phone use definition. However, it is not widely established in empirical research, and I take this to be included in the other components which all signal a loss of control.

6 The ideal size of focus groups if often found to be 5–8 participants. In an online environment such as the one for this study, fairly small groups were chosen so that participants could see each other reasonably well on the screen.

7 The extremely high usage of the word ‘like’ by students regularly made sentences incomprehensible, and so the word was deleted in most instances.

8 More specifically, students took courses in Medicine (6), Politics, Philosophy and Economics (6), Politics and IR (5), Geography (5), Law (5), Sociology (3), Biological Sciences (2), Modern Languages (1), Criminology (1), Liberal Arts (1), Health Data Science (1), History (1).

9 The sample of the survey is slightly larger than that of focus groups participants (*N* = 45) because some students could not attend the focus group due to illness.

10 In the behavioural addiction literature, it is considered that if three or more criteria are met within a 12-month period, an individual can be defined as addicted.

## Appendix 1

### Focus group questionnaire

#### Smartphone usage

What do you mostly do, with your smartphone? What makes you use your smartphone?

How much time do you usually spend on your phone?

Are you always available?

Do you find it easy or difficult to be without your smartphone?

Does your smartphone usage affect other areas of life?

How would you say the use of smartphones supports your wellbeing?

How would you say the use of smartphones undermines your wellbeing?

#### Social media usage

What do you mostly do when on social media? What makes you use social media?

How much time do you usually spend on social media?

Do you find it easy or difficult to not be on social media?

Do you ever experience fear of missing out?

Do you find yourself comparing yourself to others, on social media?

Do you use image editing/filters when posting?

How would you say the use of social media supports your wellbeing?

How would you say the use of social media undermines your wellbeing?

#### Exit questions

- Is there anything we did not touch on that you feel is important?
- Is there a specific topic we want to circle back to from this discussion to add or expand on?

## Appendix 2

### Survey items

- Q1-Gender: how do you identify?
- Q2-How old are you?
- Q3-How old were you when you got your first smartphone?
- Q4-How old were you when you created your first social media account?
- Q5-How many social media accounts do you have?
- Q6-What is your daily screen time?
- Q7-How much of your daily time do you spend on social media?
- Q8-Which applications do you use the most on your smartphone? Rank them from most used to least used.
- Q9-It is very easy for me not to use social media for 24 h.
- Q10-It is very easy for me not to use my smartphone for 24 h.

## References

[ref1] Abeele M. V. de Wolf R. Ling R. (2018). Mobile media and social space: how anytime, anyplace connectivity structures everyday life. Media Commun. 6, 5–14. doi: 10.17645/mac.v6i2.1399

[ref2] Aksoy M. E. (2018). A qualitative study on the reasons for social media addiction. Eur. J. Educ. Res. 7, 861–865. doi: 10.12973/eu-jer.7.4.861

[ref3] Alter A. (2017). Irresistible: The Rise of Addictive Technology and the Business of Keeping Us Hooked. New York: Penguin.

[ref4] Andreassen C. S. Pallesen S. Griffiths M. D. (2017). The relationship between addictive use of social media, narcissism and self-esteem: findings from a large national survey. Addict. Behav. 64, 287–293. doi: 10.1016/j.addbeh.2016.03.006, 27072491

[ref5] Baumeister R. Leary M. R. (1995). The need to belong. Psychol. Bull. 117, 497–529. doi: 10.1037/0033-2909.117.3.497, 7777651

[ref6] Billieux J. Maurage P. Lopez-Fernandez O. Kuss D. J. Griffiths M. D. (2015a). Can disordered mobile phone use be considered a behavioral addiction? An update on current evidence and a comprehensive model for future research. Curr. Addict. Rep. 2, 156–162. doi: 10.1007/s40429-015-0054-y

[ref7] Billieux J. Philippot P. Schmid C. Maurage P. de Mol J. van der Linden M. (2015b). Is dysfunctional use of the Mobile phone a behavioural addiction? Confronting symptom-based versus process-based approaches. Clin. Psychol. Psychother. 22, 460–468. doi: 10.1002/cpp.1910, 24947201

[ref8] Billieux J. Van der Linden M. Rochat L. (2008). The role of impulsivity in actual and problematic use of the mobile phone. Appl. Cogn. Psychol. 22, 1195–1210. doi: 10.1002/acp.1429

[ref9] Braun V. Clarke V. (2006). Using thematic analysis in psychology. Qual. Res. Psychol. 3, 77–101. doi: 10.1191/1478088706qp063oa

[ref10] Carey M. A. (1994). “The group effect in focus groups: planning, implementing and interpreting focus group research,” in Critical Issues in Qualitative Research Methods, ed. Morse J. (Thousand Oaks: Sage), 225–241.

[ref11] Carvalho L. F. Sette C. P. Ferrari B. L. (2018). Problematic smartphone use relationship with pathological personality traits: systematic review and meta-analyzes. Cyberpsychology 12:5. doi: 10.5817/CP2018-3-5

[ref12] Cheng J. Chen Y. Liu J. Gao S. Ju Y. Liu B. . (2025). Perfectionism, obsessive-compulsory behaviour, and anxiety in young adults: a moderated mediation model of mobile phone addiction. BMC Public Health 25:1–9. doi: 10.1186/s12889-025-24119-8, 40830458 PMC12363133

[ref13] Conroy D. Chadwick D. Fullwood C. Lloyd J. (2023). You have to know how to live with it without getting to the addiction part’: British young adult experiences of smartphone over-reliance and dysconnectivity. Psychol. Pop. Media 12, 471–480. doi: 10.1037/ppm0000425

[ref14] David M. E. Roberts J. A. (2017). Phubbed and alone: phone snubbing, social exclusion, and attachment to social media. J. Assoc. Consum. Res. 2, 155–163. doi: 10.1086/690940

[ref15] Foucault M. (1977). The knowledge of power and the power of knowledge. Bull. Hist. Med. 51, 245–277. 329924

[ref16] Francis J. Francis J. J. Johnston M. Robertson C. Glidewell L. Entwistle V. . (2010). What is an adequate sample size? Operationalising data saturation for theory-based interview studies. Psychol. Health 25, 1229–1245. doi: 10.1080/08870440903194015, 20204937

[ref17] Friedrichs J. Kratochwil F. (2009). On acting and knowing: how pragmatism can advance international relations research and methodology. Int. Organ. 63, 701–731. doi: 10.1017/s0020818309990142

[ref18] Fullwood C. Quinn S. Kaye L. K. Redding C. (2017). My virtual friend: a qualitative analysis of the attitudes and experiences of smartphone users: implications for smartphone attachment. Comput. Hum. Behav. 75, 347–355. doi: 10.1016/j.chb.2017.05.029

[ref19] Griffiths M. D. (2005). A ‘components’ model of addiction within a biopsychosocial framework. J. Subst. Use 10, 191–197. doi: 10.1080/14659890500114359

[ref21] Ha Y. W. Kim J. Libaque-Saenz C. F. Chang Y. Park M.-C. (2015). Use and gratifications of mobile SNSs: Facebook and KakaoTalk in Korea. Telemat. Inform. 32, 425–438. doi: 10.1016/j.tele.2014.10.006

[ref22] Hadlington L. J. (2015). Cognitive failures in daily life: exploring the link with internet addiction and problematic mobile phone use. Comput. Hum. Behav. 51, 75–81. doi: 10.1016/j.chb.2015.04.036

[ref23] Han S. Kim K. J. Kim J. H. (2017). Understanding nomophobia: structural equation modelling and semantic network analysis of smartphone separation anxiety. Cyberpsychol. Behav. Soc. Netw. 20, 419–427. doi: 10.1089/cyber.2017.0113, 28650222

[ref24] Hancock M. Amankwaa L. Revell M. Mueller D. (2016). Focus group data saturation: a new approach to data analysis. Qual. Rep. 21, 2121–2130. doi: 10.46743/2160-3715/2016.2330, 39386316

[ref25] Harkin L. J. Kuss D. (2021). “My smartphone is an extension of myself”: a holistic qualitative exploration of the impact of using a smartphone. Psychol. Pop. Media 10, 28–38. doi: 10.1037/ppm0000278

[ref26] Hong W. Liu R. D. Ding Y. Sheng X. Zhen R. (2020). Mobile phone addiction and cognitive failures in daily life: the mediating roles of sleep duration and quality and the moderating role of trait self-regulation. Addict. Behav. 107:106383. doi: 10.1016/j.addbeh.2020.106383, 32200196

[ref27] Horwood S. Anglim J. (2021). Emotion regulation difficulties, personality, and problematic smartphone use. Cyberpsychol. Behav. Soc. Netw. 24, 275–281. doi: 10.1089/cyber.2020.0328, 33090002

[ref28] Hunt M. G. Marx R. Lipson C. Young J. (2018). No more FOMO: limiting social media decreases loneliness and depression. J. Soc. Clin. Psychol. 37, 751–768. doi: 10.1521/jscp.2018.37.10.751

[ref29] Jameel S. Ghazi Shahnawaz M. Griffiths M. (2019). Smartphone addiction in students: a qualitative examination of the components model of addiction using face-to-face interviews. J. Behav. Addict. 8, 780–793. doi: 10.1556/2006.8.2019.57, 31619046 PMC7044586

[ref30] Kardefelt-Winther D. Heeren A. Schimmenti A. van Rooij A. Maurage P. Carras M. . (2017). How can we conceptualize behavioural addiction without pathologizing common behaviours? Addiction 112, 1709–1715. doi: 10.1111/add.13763, 28198052 PMC5557689

[ref31] Kidd P. S. Parshall M. B. (2000). Getting the focus and the group: enhancing analytical rigor in focus group research. Qual. Health Res. 10, 293–308. doi: 10.1177/104973200129118453, 10947477

[ref32] King A. L. S. Valença A. M. Silva A. C. O. Baczynski T. Carvalho M. R. Nardi A. E. (2013). Nomophobia: dependency on virtual environments or social phobia? Comput. Hum. Behav. 29, 140–144. doi: 10.1016/j.chb.2012.07.025

[ref33] Kitzinger J. (1994). The methodology of focus groups: the importance of interaction between research participants. Sociol. Health Ill. 16, 103–121. doi: 10.1111/1467-9566.ep11347023, 40046247

[ref34] Kitzinger J. (1995). Qualitative research: introducing focus groups. Br. Med. J. 311, 299–302. doi: 10.1136/bmj.311.7000.299, 7633241 PMC2550365

[ref35] Konok V. Gigler D. Bereczky B. M. Miklósi Á. (2016). Humans' attachment to their mobile phones and its relationship with interpersonal attachment style. Comput. Hum. Behav. 61, 537–547. doi: 10.1016/j.chb.2016.03.062

[ref36] Konok V. Pogany Á. Miklósi Á. (2017). Mobile attachment: separation from the mobile phone induces physiological and behavioural stress and attentional bias to separation-related stimuli. Comput. Hum. Behav. 71, 228–239. doi: 10.1016/j.chb.2017.02.002

[ref37] Kuss D. (2017). Mobile technology and social media: the “extensions of man” in the 21st century. Hum. Dev. 60, 141–143. doi: 10.1159/000479842

[ref38] Kuss D. Griffiths M. D. (2017). Social networking sites and addiction: ten lessons learned. Int. J. Environ. Res. Public Health 14:311. doi: 10.3390/ijerph14030311, 28304359 PMC5369147

[ref39] Kuss D. Harkin L. Kanjo E. Billieux J. (2018). Problematic smartphone use: investigating contemporary experiences using a convergent design. Int. J. Environ. Res. Public Health 15, 142–142116. doi: 10.3390/ijerph15010142, 29337883 PMC5800241

[ref40] Kwon M. Lee J. Y. Won W. Y. Park J. W. Min J. A. Hahn C. . (2013). Development and validation of a smartphone addiction scale (SAS). PLoS One 8:e56936. doi: 10.1371/journal.pone.0056936, 23468893 PMC3584150

[ref41] Li L. Niu Z. Mei S. Griffiths M. D. (2022). A network analysis approach to the relationship between fear of missing out (FoMO), smartphone addiction, and social networking site use among a sample of Chinese university students. Comput. Hum. Behav. 128:1–10. doi: 10.1016/j.chb.2021.107086

[ref42] Mai L. M. Freudenthaler R. Schneider F. M. Vorderer P. (2015). “I know you’ve seen it!” individual and social factors for users' chatting behavior on Facebook. Comput. Hum. Behav. 49, 296–302. doi: 10.1016/j.chb.2015.01.074

[ref43] Marengo D. Sindermann C. Häckel D. Settanni M. Elhai J. D. Montag C. (2020). The association between the big five personality traits and smartphone use disorder: a meta-analysis. J. Behav. Addict. 9, 534–550. doi: 10.1556/2006.2020.00069, 33011714 PMC8943667

[ref44] Matar Boumosleh J. Jaalouk D. (2017). Depression, anxiety, and smartphone addiction in university students - a cross sectional study. PLoS One 12:1–14. doi: 10.1371/journal.pone.0182239, 28777828 PMC5544206

[ref45] Montoya-Weiss M. M. Massey A. P. Clapper D. L. (1998). Online focus groups: conceptual issues and a research tool. Eur. J. Mark. 32, 713–723. doi: 10.1108/03090569810224100

[ref46] Morgan D. L. (1996). Focus groups. Annu. Rev. Sociol. 22, 129–152. doi: 10.1146/annurev.soc.22.1.129

[ref47] Olivencia-Carrión M. A. Ferri-García R. Rueda M. M. Jiménez-Torres M. G. López-Torrecillas F. (2018). Temperament and characteristics related to nomophobia. Psychiatry Res. 266, 5–10. doi: 10.1016/j.psychres.2018.04.056, 29787807

[ref48] Perrin A. Anderson M. (2019). Share of US Adults Using Social Media, Including Facebook, is Mostly Unchanged since 2018. Washington D.C.: Pew Research Center.

[ref49] Pokhrel P. Herzog T. A. Black D. S. Zaman A. Riggs N. R. Sussman S. (2013). Adolescent neurocognitive development, self-regulation, and school-based drug use prevention. Prev. Sci. 14, 218–228. doi: 10.1007/s11121-012-0345-7, 23408284 PMC4181554

[ref50] Pontes H. M. Taylor M. Stavropoulos V. (2018). Beyond “Facebook addiction”: the role of cognitive-related factors and psychiatric distress in social networking site addiction. Cyberpsychol. Behav. Soc. Netw. 21, 240–247. doi: 10.1089/cyber.2017.0609, 29589972

[ref51] Przybylski A. K. Murayama K. DeHaan C. R. Gladwell V. (2013). Motivational, emotional, and behavioral correlates of fear of missing out. Comput. Hum. Behav. 29, 1841–1848. doi: 10.1016/j.chb.2013.02.014

[ref52] Reer F. Tang W. Y. Quant T. (2019). Psychosocial well-being and social media engagement: the mediating roles of social comparison orientation and fear of missing out. New Media Soc. 21, 1486–1505.

[ref53] Reiner I. Tibubos A. N. Hardt J. Müller K. Wölfling K. Beutel M. E. (2017). Peer attachment, specific patterns of internet use and problematic internet use in male and female adolescents. Eur. Child Adolesc. Psychiatry 26, 1257–1268. doi: 10.1007/s00787-017-0984-0, 28378129

[ref54] Ryding F. C. Kaye L. K. (2018). ‘Internet addiction’: a conceptual minefield. Int. J. Ment. Heal. Addict. 16, 225–232. doi: 10.1007/s11469-017-9811-6, 29491771 PMC5814538

[ref55] Samaha M. Hawi N. S. (2016). Relationships among smartphone addiction, stress, academic performance, and satisfaction with life. Comput. Hum. Behav. 57, 321–325.

[ref56] Throuvala M. A. Griffiths M. D. Rennoldson M. Kuss D. J. (2019). Motivational processes and dysfunctional mechanisms of social media use among adolescents: a qualitative focus group study. Comput. Hum. Behav. 93, 164–175. doi: 10.1016/j.chb.2018.12.012

[ref57] Turkle S. (2017). Alone Together: Why we Expect more from Technology and less from each other. London: Hachette.

[ref58] Vorderer P. Klimmt C. (2020). “The Mobile’s user mindset in a permanently online, permanently connected society,” in The Oxford Handbook of Mobile Communication and Society, eds. Ling R. . (Oxford: Oxford University Press), 54–67.

[ref59] Walsh S. P. White K. M. Young R. M. (2008). Over-connected? A qualitative exploration of the relationship between Australian youth and their mobile phones. J. Adolesc. 31, 77–92. doi: 10.1016/j.adolescence.2007.04.004, 17560644

[ref60] Walz S. P. Deterding S. (2015). The Gameful World: Approaches, Issues, Applications. Boston: MIT Press.

[ref61] Yildirim C. Correia A.-P. (2015). Exploring the dimensions of nomophobia: development and validation of a self-reported questionnaire. Comput. Hum. Behav. 49, 130–137. doi: 10.1016/j.chb.2015.02.059

[ref63] Ytre-Arne B. Syvertsen T. Moe H. Karlsen F. (2020). Temporal ambivalences in smartphone use: conflicting flows, conflicting responsibilities. New Media Soc. 22, 1715–1732. doi: 10.1177/1461444820913561

[ref64] Zuboff S. (2019). The Age of Surveillance Capitalism: The Fight for a Human Future at the New Frontier of Power. New York: Public Affairs.

